# A Subdomain Method for Mapping the Heterogeneous Mechanical Properties of the Human Posterior Sclera

**DOI:** 10.3389/fbioe.2019.00129

**Published:** 2019-05-31

**Authors:** Hirut G. Kollech, Avinash Ayyalasomayajula, Reza Behkam, Ehab Tamimi, Kenneth Furdella, Michelle Drewry, Jonathan P. Vande Geest

**Affiliations:** ^1^Computational Modeling and Simulation Program, University of Pittsburgh, Pittsburgh, PA, United States; ^2^Department of Aerospace and Mechanical Engineering, University of Arizona, Tucson, AZ, United States; ^3^Department of Bioengineering, University of Pittsburgh, Pittsburgh, PA, United States; ^4^McGowan Institute for Regenerative Medicine, University of Pittsburgh, Pittsburgh, PA, United States; ^5^Department of Ophthalmology, University of Pittsburgh, Pittsburgh, PA, United States; ^6^Louis J. Fox Center for Vision Restoration, University of Pittsburgh, Pittsburgh, PA, United States

**Keywords:** glaucoma, sclera, inverse finite element, optic nerve head, subdomain approach

## Abstract

Although strongly correlated with elevated intraocular pressure, primary open-angle glaucoma (POAG) occurs in normotensive eyes. Mechanical properties of the sclera around the optic nerve head (ONH) may play a role in this disparity. The purpose of this study is to present an automated inverse mechanics based approach to determine the distribution of heterogeneous mechanical properties of the human sclera as derived from its surface deformations arising from pressure inflation experiments. The scleral shell of a 78 year old European Descent male donor eye was utilized to demonstrate the method; the sclera was coated with a speckle pattern on the outer surface and was subjected to inflation pressures of 5, 15, 30, and 45 mmHg. The speckle pattern was imaged at each pressure, and a displacement field was calculated for each pressure step using a previously described sequential digital image correlation (S-DIC) technique. The fiber splay and fiber orientation of the sclera collagen were determined experimentally, and the thickness across the scleral globe was determined using micro CT images. The displacement field from the inflation test was used to calculate the strain and also used as an input for inverse mechanics to determine the heterogeneity of material properties. The scleral geometry was divided into subdomains using the first principal strain. The Holzapfel anisotropic material parameters of matrix and fiber stiffness were estimated within each individual subdomain using an inverse mechanics approach by minimizing the sum of the square of the residuals between the computational and experimental displacement fields. The mean and maximum error in displacement across all subdomains were 8.9 ± 3.0 μm and 13.2 μm, respectively. The full pressure-inflation forward mechanics experiment was done using subdomain-specific mechanical properties on the entire scleral surface. The proposed approach is effective in determining the distribution of heterogeneous mechanical properties of the human sclera in a user-independent manner. Our research group is currently utilizing this approach to better elucidate how scleral stiffness influences those at high risk for POAG.

## Introduction

The ocular system is a combination of several micro- and macroscopic tissues working in harmony to sense visual information and transfer it to the brain via the optic nerve. Like many ocular pathologies, primary open-angle glaucoma (POAG) leads to an irreversible damage to the cells responsible for transducing the visual signals. Glaucoma is projected to be the leading cause of blindness, second only to cataracts, affecting a significant percentage of populations across different ages and race/ethnic groups (Quigley and Broman, 2006; Rudnicka et al., 2006). A hallmark of POAG is the severe loss of retinal ganglion cells (RGCs) (Almasieh et al., 2012). These cells line the inner surface of the retina and carry visual information from the eye to the brain. In a normal human eye, the mean intraocular pressure (IOP) is approximately 15 mmHg. There is evidence that elevated IOP leads to “abnormal extracellular matrix [ECM] remodeling in the retina” and consequently the dysfunction of RGCs (Guo et al., 2017). Similar degradation of RGCs also occurs in patients with normal tension glaucoma, indicating that factors other than elevated IOP likely play a role in the disease (Drance et al., 2001). Of particular importance with regard to the incidence of POAG is the lamina cribrosa (LC)—a highly porous tissue that surrounds the nerve fibers in the optic nerve head (ONH). Structural changes in this tissue have been previously associated with glaucoma, both *in vivo* and *in vitro* (Drance et al., 2001). Also, tissues in and around the ONH, which include the LC and the peripapillary sclera, have shown to influence the biomechanical response of the ONH through their stiffness and permeability (Tengroth et al., 1985; Morgan et al., 1995, 1998; Downs et al., 2003; Sigal et al., 2005; Girard et al., 2009; Grytz et al., 2014a; Coudrillier et al., 2015b) For example, a stiffer sclera has been shown to reduce strains in the LC (Sigal et al., 2004, 2005; Thornton et al., 2009), and a low permeability of the retina-Bruch's-choroid complex increases LC strains (Ayyalasomayajula et al., 2010). These studies have hypothesized that the biomechanics of the tissues in the ONH region could play an important role in the incidence of POAG.

Previous studies have shown that the mechanical properties of human ocular tissue vary with location in the tissue (Elsheikh et al., 2007; Coudrillier et al., 2012). Collagen orientation in the sclera varies from significantly aligned in the peripapillary region to less aligned toward the equator (Coudrillier et al., 2012) with stiffness decreasing from the anterior to the posterior eye (Friberg and Lace, 1988). Scleral collagen fiber orientation was shown to vary with age, race, and ethnicity (Yan et al., 2011a; Danford et al., 2013), with its stiffness being found to decrease with age in an experimental study (Geraghty et al., 2012). Some previous computational studies have incorporated anisotropy and microstructurally-based constitutive models to study the biomechanical response of peripapillary sclera (Girard et al., 2009; Grytz and Meschke, 2009). Recently, vibrational analysis has also been utilized to estimate the mechanical properties of ocular tissues (Aloy et al., 2017). Studies like these may be important in elucidating the mechanisms by which certain populations are more highly predisposed to POAG than others (Kroese et al., 2002; Rudnicka et al., 2006).

To explore this hypothesis, the focus of some recent studies has been to characterize material anisotropy and heterogeneity in the posterior scleral shell (Girard et al., 2009; Coudrillier et al., 2012; Grytz et al., 2014b) using the inverse mechanics technique. In this technique, scleral tissue is inflated to varying pressures, and data on surface deformations, orientation and splay of collagen fibers are recorded using techniques like wide angle X-ray scattering (WAXS) (Coudrillier et al., 2012, 2015a) and small angle light scattering (SALS) (Yan et al., 2011b). A computational mesh of the sclera is generated, and the experimental conditions (fixed base and pressure on the inner scleral surface) are imposed as boundary conditions. A constitutive model is chosen *a priori*, and the model parameters are estimated using an optimization algorithm to minimize a cost function which involves the surface deformation information of the inflated sclera. Depending on the complexity of the constitutive model, there could be multiple parameters involved in the optimization process. Simultaneously fitting multiple model parameters (which can vary with location in the sclera) for the entire scleral domain can involve heavy computational cost. While dividing the scleral domain into regularly spaced equatorial and meridional regions could reduce the computational effort, it may lead to subdomains that span regions with different material properties and could lead to an inaccurate estimation of model parameter values. To address this issue, we herein describe a technique to adaptively divide the domain of the scleral shell into multiple subdomains based on the experimental surface deformation data. This technique results in distinct regions (which we refer to as “subdomains”) for which the material properties are assumed to be uniform. An optimization algorithm is then applied to determine the material model parameters of each subdomain. The parallelizable nature of the proposed approach minimizes computational time while also being adaptive enough to capture a wide variety of spatial distributions, from homogenous to highly heterogeneous, all in a user independent manner.

## Methods

In the current study, a posterior sclera shell from a 78 year old European Descent male donor has been utilized. The eye was acquired within 48-h post-mortem from Michigan Eye Bank (Eversight) in Ann Arbor, MI, USA. The procedures in this study adhered to the tenets of the Declaration of Helsinki. The consent procedure used for obtaining the specimen was in accordance with the Revised Uniform Anatomical Gift Act (2006), Michigan Uniform Anatomical Gift Law and Michigan Revised Uniform Anatomical Gift Law. The eye bank also obtained all ethical approval to use the specimens for research purposes. The sample was used to demonstrate the technique of estimating the material property distribution. In the following sections, the various aspects of this technique have been described.

### Sclera Geometry and Displacement Field Data

Our laboratory has previously presented a sequential digital image correlation (S-DIC) technique to quantify surface deformation of human ocular posterior poles (Pyne et al., 2014). Briefly, the eye globe was cut along the hemisphere, centering the ONH, and the retina, choroid and vitreous humor were removed from the inside of the posterior shell. The sample was kept hydrated with 1xPBS throughout this dissection procedure. In order to maintain the posterior sclera in its physiological shape without any leaks, custom lightweight clamps were designed. These clamps have an inlet and outlet for saline pressurization of the inner scleral surface and clearance holes for calibration. Any excess sclera that did not fit into the clamps was removed. After clamping, the sclera was sprayed with black and white ink to create speckle pattern that was later used to perform DIC matching between image pairs. It was then moved into a humidity chamber that was already at 37°C and 95% humidity and was left for the ink to settle properly. The clamps were attached to rotary stages, a calibration cone and an automated pressure regulation system. The pressure was regulated using a pressure transducer (Omega PX309002GV ± 0.26 mmHg) and actuated using a programmable syringe pump (New Era Pump Systems, Inc. NE-1000) that was controlled through a Labview (National Instruments) script (Pyne et al., 2014). The sclera was initially inflated to 5 mmHg (reference state) and subsequently inflated to pressures of 15, 30, and 45 mmHg (final deformed state). After applying pressure, there was a 10-min wait time to decrease the creep effect before taking displacement measurements (Downs et al., 2003; Pyne et al., 2014). At each pressure step, the sample was imaged using a Siemens Inveon scanner (microcomputer tomograph) with the following parameters: 80 kVp, 450 μA, 1,650 ms exposure, 220 degrees of rotation, a binning of 2, and a 53.79 transaxial by 53.79 axial mm field of view. The voxel size was 35.6 μm. A digital camera was used to capture the images of the speckle pattern on the scleral surface in the reference and deformed states. A set of evenly spaced images were extracted and used to reconstruct a 3D point cloud of the surface and displacement was calculated from the point cloud. For each of the pressure steps, the imaging was split into four large stereo-angle sweeps that covered meridional lines starting from the center of the ONH, in the nasal, temporal, superior and inferior directions, with the lines ending at the base of the sclera. The S-DIC approach was used to track the position of speckles in each sweep at each pressure state. The 3D point cloud from each of the four sweeps for a given pressure were then combined to assemble the scleral and ONH geometry (Tamimi et al., 2017). The ONH was removed and the cross-sections of the points on the sclera at different segments were created in the sagittal view. These points were imported to SOLIDWORKS® and using the loft feature, a smooth shell geometry for the finite element simulation was generated.

### Thickness

**Figure 1:**
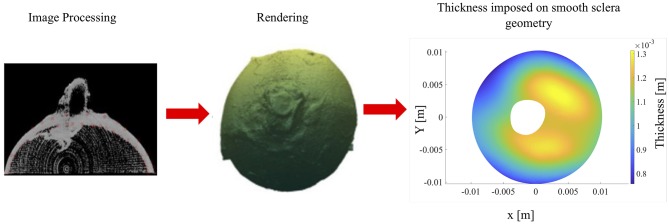
Sclera thickness: thickness calculation of the sclera shell from micro CT images **(Left)** to reconstructed geometries (triangular meshes) of the interior and exterior scleral surfaces **(Middle)**, to the final imposed thickness on smooth sclera geometry and its distribution across the sclera globe **(Right)**.

The sclera thickness was determined from a micro CT image of the sample. The image sets in the reference state (5 mmHg) were thresholded to remove background noise. Next, points were manually selected from the interior and exterior boundaries of the sclera. These point clouds were then separated to form contiguous and separate interior and exterior meshes which were then smoothed. For the thickness calculation, each normal from every element was projected onto the smoothed interior point cloud, and three closest points were identified. These three points were fit to a plane, and the distance from the exterior element and the fit plane were used to calculate thickness. The thickness values were then plotted onto each element on the exterior mesh to give a visual representation of scleral thickness; it had a mean thickness of 1.065 ± 0.147 mm (see Figure 1). Finally, the optic nerve head was removed, and the sclera was divided into four regions (superior, inferior, nasal and temporal).

### Material Constitutive Model

Scleral tissue has previously been shown to be anisotropic (Girard et al., 2009; Coudrillier et al., 2015b; Pijanka et al., 2015). For the present work, we chose to use the Holzapfel model (Holzapfel et al., 2000) to capture anisotropic constitutive behavior of scleral tissue. However, the approach presented is flexible enough to be easily adapted to a wide variety of anisotropic constitutive models. The strain energy function, as implemented in ABAQUS (Dassualt Systèmes (ed.)., 2011), is given by:

$$\begin{array}{l} W=C_{10\;}\left(\overset{-}{I_{1}}-3\right)+\frac{k_{1}}{2k_{2}}\overset{N}{\underset{\alpha =1}{\sum }}\left\{\exp\left[k_{2}{\langle E_{\alpha }\rangle }^{2}\right]-1\right\} \end{array}$$

where,

$$E_{\alpha }=\kappa \;\left(\overset{-}{I_{1}}-3\right)+\left(1-3\kappa \right)\left(\overset{-}{I_{4}}-1\right),\text{and }\overset{-}{I_{4}}={\text{A}}_{\alpha }.\overset{-}{\text{C}}.{\text{A}}_{\alpha }$$

κ, *C*_10_, κ_1_, and κ_2_ are material parameters, and we have assumed one fiber family is in the sclera (*N* = 1). The parameter “κ ” describes the fiber dispersion about the mean fiber orientation at any given location: κ = 0 for perfect alignment and $\kappa =\frac{1}{3}$ for random distribution. $\overset{-}{I_{4}}$is the pseudo-invariant of the modified right Cauchy-Green tensor, $\overset{-}{\text{C}}$, and **A**_α_ denotes the unit vector in the direction of the fiber. The sclera was assumed to be incompressible in the current work. The parameter “κ” and the angle of the scleral collagen were experimentally determined using the small angle light scattering (SALS) technique (Danford et al., 2013) and the κ values were 0.249, 0.287, 0.248, and 0.170 for subdomains 1 through 4, respectively.

### Subdomains

**Figure 2:**
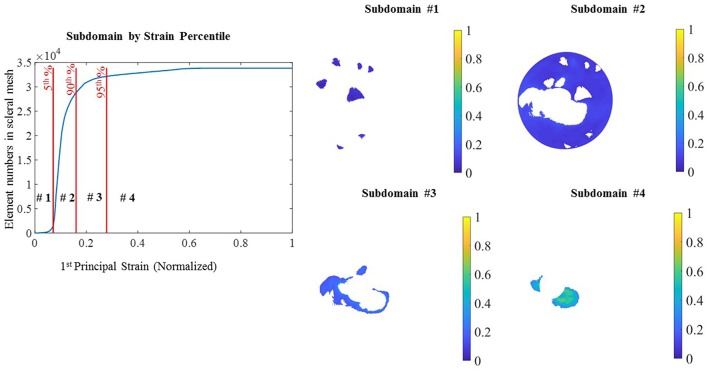
Subdomain from strain: subdomains were generated from the 1st principal strain percentile. Normalized strain values in ascending order in the sclera elements with the different percentile **(Left)** and 4 subdomains generated from the percentile ranges.

**Figure 3:**
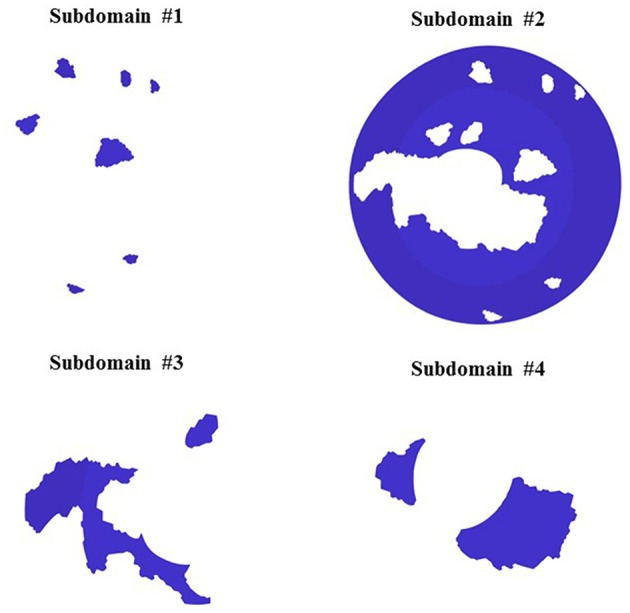
Final Subdomains and mesh for inverse finite simulation.

For the optimization process, the entire scleral surface was divided into subdomains. This division was made on the basis of the magnitude of the sclera first principal strain (E1) when it was pressure-inflated from the reference state (5 mmHg) to a deformed state (45 mmHg) by cumulating the displacements from all pressure steps. To generate the subdomains based on strain maps, the reconstructed point clouds at 5 mmHg and 45 mmHg were used to create a geometry mesh. The Green–Lagrange strain tensor, E, was computed for each element using the equation, *E* = 0.5 (F^T^F-I), where F is the deformation gradient tensor and I is the identity matrix. *E* was used to calculate first principal strains for each element. Additional details of the approach can be found in (Pyne et al., 2014; Tamimi et al., 2017). The intermediary pressure set measurements (15 and 30 mmHg) were utilized for the Finite element simulation and optimization. First the displacement field was smoothed using Gibbons smoothing function in MATLAB (Moerman, 2018). Then this displacement was used to calculate principal strains. The strain magnitude was normalized, and percentiles of the normalized strain (E1) were used to divide the sclera into subdomains. In Figure 2 the initial subdomains were generated based on the strain maps. However, in the rare occasion that a single layer of elements was surrounded by two regions (for example the long strip in subdomain 3) a MATLAB code was developed to assign the elements in the single layer region to the neighboring subdomain. When assigning a single layer region to a subdomain, if it has two neighboring subdomains, the difference between the strain values of surrounding elements in the neighboring subdomains and the single layer region were compared and the one with the smaller strain difference was assigned to be the neighboring subdomain. This criterion was implemented in the MATLAB code. The final subdomains created after this step are shown in Figure 3.

### Computational Mesh and Boundary Condition

**Figure 4:**
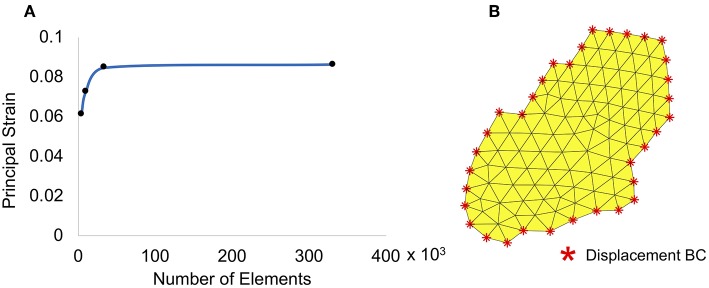
**(A)** Mesh convergence and **(B)** a small subset of subdomain 3 with boundary conditions.

The scleral surface mesh was generated using linear triangular plane stress elements. A mesh convergence study was conducted to determine the size of the element that resulted in a mesh-size independent solution. Principal strain values in a small region (further away from the boundaries) were collected for the convergence study, and the results are shown in Figure 4A. Based on these results, a mesh with ~34,000 elements and a maximum size of 17 μm was used for the simulation. A representative geometric model showing displacement boundary conditions and mesh on a small subset of subdomain 3 is displayed in Figure 4B. The boundary nodes for each subdomain were identified and displacements of the speckle pattern in the pressure-inflation experiments at these locations were imposed as prescribed boundary conditions. The internal surface of each subdomain mesh was sequentially pressurized to 15, 30, and 45 mmHg; the internal nodes within each subdomain were free to displace/rotate under the above applied pressures. The displacement measurements at 15, 30, and 45 mmHg of the boundary subdomain nodes were imposed as boundary conditions.

## Optimization

**Figure 5:**
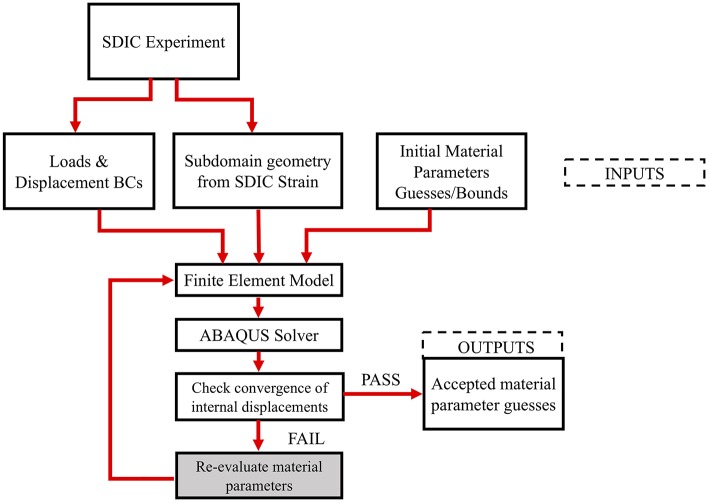
Inverse finite element optimization workflow.

The Holzapfel model parameters (*C*_10_, *k*_1_, *k*_2_), for each subdomain were determined by a MATLAB built-in derivative free unconstrained optimization algorithm (particle swarm) (Shi and Eberhart, 1999). To ensure that the solution is not a local minimum of the parameter space, a DOE was conducted on each subdomain as follows: a wide range of values were chosen for each of the unknown parameters of the Holzapfel constitutive model (5 kPa to 40 MPa for “*C*_10_,” 1 Pa to 40 MPa for “*k*_1_,” and 0.1 to 300 for “*k*_2_”), and the parameter space was discretized to generate several combinations of these values. A forward finite element (FE) analysis was performed for each of these parameter combinations, and the displacements were output at the internal nodes. Upper and lower bounds from the forward study were provided to the particle swarm algorithm, and each subdomain was assigned a new material parameter and pressurized on the inside surface. Displacement measurements of internal nodes were used to calculate the residual. The sum of the squares of the difference in the nodal displacements of the internal nodes resulting from the simulation and the experiments (at corresponding nodes and pressure states) was calculated as the residual. The optimization converges on a material parameter set that minimizes the sum of squares of the residuals. The average difference between the experimental and computational nodal displacements was calculated as follows. First, the displacement results of the computational results were collected at the interior nodes. Then at each pressure step, the difference between the computational and the experimental results were calculated using the sum of the square of the difference. Finally, the sum of these values from all pressures was divided by the total number of the interior nodes and used as the average difference. This was performed on each subdomain resulting in a heterogeneous material property distribution for the scleral shell. The flowchart for the entire process is shown in Figure 5.

Following the above optimization process on each subdomain, the individual subdomains were assembled to make the entire scleral domain, and the corresponding optimized material properties were imposed on them. The surface displacements resulting from internal pressurization through the three pressure steps were compared with the experimentally determined displacements. This step serves as a check to ensure that the estimated material parameters for each subdomain, when combined into a continuous scleral shell, results in the surface deformation from the inflation test and as such truly represent the constitutive behavior of the experimentally tested sclera. Before running the full sclera simulation, the optimized material parameters coming from the individual subdomains were merged together. From physiological point of view, abrupt variation in material properties is not expected therefore, a smoothing procedure (Moerman, 2018) helped to create a transition region between neighboring subdomains and morphed material properties from one region into other ones.

## Results

**Figure 6:**
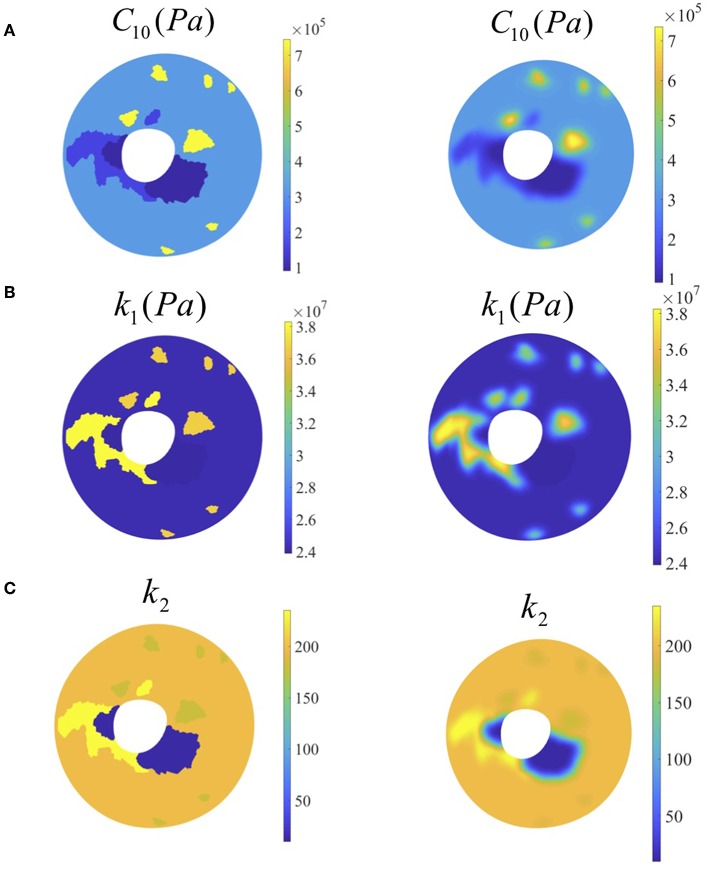
Left column: contour plots of optimized material parameter values from optimization (before smoothing). Right column: contour plots of optimized material parameter values from optimization (after smoothing) **(A)** matrix modulus stiffness, **(B)** fiber stiffness, **(C)** fiber parameter.

The displacement values after loading to the final pressure (45 mmHg) were as follows: Ux (−2.12 × 10^−5^ ± 3.54 × 10^−5^ m) with range [−1.32 × 10^−4^, 7.92 × 10^−5^] m, Uy (2.12 × 10^−5^ ± 3.25 × 10^−5^ m) with range [−1.44 × 10^−4^, 8.53 × 10^−5^] m and Uz (2.59 × 10^−5^ ± 1.17 × 10^−4^ m) with range [−2.24 × 10^−4^, 5.60 × 10^−4^] m. In addition, our results show that the distribution of the matrix modulus parameter varied between 0.09 and 0.75 MPa, the fiber stiffness parameter varied between 23.9 and 38.3 MPa and fiber material constant varied between 9.7 and 235.3 (see Figure 6). The average difference between the nodal displacement obtained from the experimental data (S-DIC) and the computational counterparts in all the subdomains was 8.9 × 10^−6^ ± 3.0 × 10^−6^ m with range [5.9 × 10^−6^, 11.9 × 10^−6^ m].

**Figure 7:**
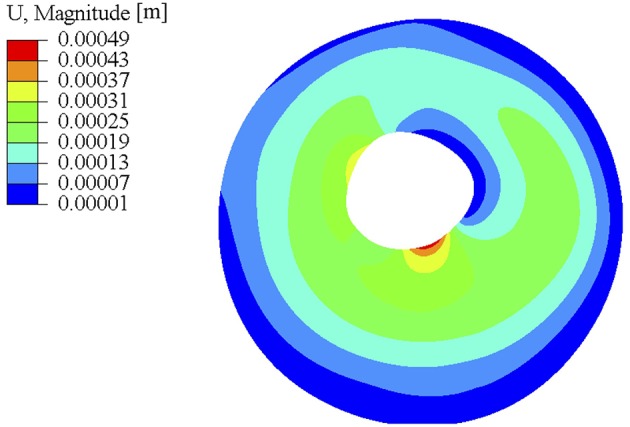
Full sclera geometry simulation using the optimized material parameters after smoothing. The material parameters used for this simulation came from subdomain results that were smoothed at the boundaries shown in the right column of Figure 6.

The distribution of *k*_2_ showed two orders of magnitude difference while the two parameters did not show such variation. A final global check simulation of the pressure-inflation was performed with all the subdomains combined and with each subdomain being assigned the estimated material properties for that region (see Figure 7). The nodal displacements output from this simulation were compared with the corresponding experimental displacements. Average run time for each forward subdomain simulation was 2 min and the entire optimization process took approximately 10 h (multiple subdomains being optimized simultaneously) on a Windows 10 workstation with 512 GB RAM and Intel Xeon CPU E5-2698 processor.

## Discussion

### Relation to Previous Work

Previously, inverse mechanics based techniques were used to determine the heterogeneity in the sclera shell (Girard et al., 2009; Coudrillier et al., 2012, 2015b; Grytz et al., 2014a,b). In some cases, the local fiber orientation was determined experimentally, and the scleral thickness was imposed by measuring the thickness at 20 points spread across the sclera surface using an ultrasound scanner (Coudrillier et al., 2015b). Girard et al. reported a method of estimating scleral heterogeneity using a complex anisotropic constitutive model that additionally outputs the local collagen orientations (Girard et al., 2009; Coudrillier et al., 2012). One difference between the method presented here and the above two studies is the way in which the sclera domain is divided into subdomains. In addition, those scleral domain methods determine the material properties of sclera either by performing the inverse mechanics technique after dividing the scleral shell into well-defined sectors at regular angular intervals or along each point on the entire sclera, with material properties of all regions determined simultaneously. In the former method, it may be possible that adjacent subdomains can end up with the same material parameters despite undergoing the optimization process separately and thus could significantly increase the computational time. In the latter method, the design space for the material properties is extremely large which greatly increases the computational effort. Although our method has a similar approach, it differs in the way the subdomains are determined and in the constitutive model chosen. Dividing the scleral domain based on the strain field reduces the number of subdomains for the optimization process, which reduces the computational time, and uses a simpler constitutive model, with 3 unknown parameters assumed to be constant within a subdomain which reduces the design space for the optimization process. The flexibility in the method allows the user to choose different material models for individual subdomains in order to achieve the experimental-measured displacements in those subdomains.

### Interpretations and Clinical Implications

The current inverse-mechanics based technique aids in better understanding the heterogeneity in the scleral shell. Our result showed that the matrix and fiber stiffness values are smaller in the subdomain region with higher strain. The overall variations in *C*_10_ and *k*_1_ values across subdomains were smaller compared to that of the parameter constant, *k*_2._ The material parameters results were comparable to a previous study done by Courdillier et al. The material properties reported in Courdillier et al across 7 human eyes had matrix shear modulus (μ) 180.71 ± 55.41 kPa (which equates to a matrix modulus of 90.36 ± 27.7 kPa) and axial fiber stiffness (4αβ) of 5.55 ± 3.43 MPa (Coudrillier et al., 2015a). One of the differences between the Courdillier et al experimental approach and our work is that Courdillier et al fitted the experimentally measured displacement to a constitutive model. In addition, the wait time post loading in their experiment was 15 min while ours was 10 min. On the other hand, it was reported previously that vision loss due to POAG varies with age and race/ethnicity (Rudnicka et al., 2006). The current method could be used to obtain useful information regarding the material property differences, if any, and the variations in their distributions across the scleral globe, helping better elucidate factors other than IOP that could be contributing to disease pathogenesis. Knowledge of heterogeneity can also help identify the reasons for asymmetric vision loss (Araie, 1995). Previous research has shown that increasing the stiffness of the sclera can reduce the strains in the LC (Sigal et al., 2004, 2005; Thornton et al., 2009). Patient-specific scleral stiffness, combined with the predictive knowledge of the scleral strains, could potentially be beneficial in a targeted approach (e.g., using topical drugs) to alter the scleral stiffness and mediate the strains in the LC to avoid further deterioration of vision in normotensive eyes or in hypertensive eyes in combination with IOP-based treatments.

### Limitations of the Technique

Despite the above advantages, the current method has some limitations. With regards to the scleral fiber architecture, it has been shown previously that there is a depth-dependent variation of the scleral collagen orientation which has been lumped into a single mean fiber angle and splay at a given location on the sclera. A full 3D model of the scleral shell to incorporate this information would be an improvement over the current method, and this is currently an area of focus within our lab. Another limitation is that the sclera geometry from S-DIC was simplified by using cross section points at different height. This was done to remove abnormal points in the raw data which would create distorted elements in the finite element analysis. Future work will explore to make the approach robust using the full geometry point clouds. Lastly, the technique presented here does not account for any prestress in the scleral shell. This could introduce some level of inaccuracy in the material parameter values. However, the relative variations between the material property values between the individual subdomains would still be a close approximation of the true heterogeneity in the scleral shell.

To our knowledge, none of the previously reported inverse-mechanics based techniques used strain as a metric to divide the scleral shell in order to estimate the material property distribution. Our method greatly reduces the computational time while providing a good approximation, evidenced by the small errors in the displacement differences, to the *in-vitro* behavior of the sclera under pressure-inflation. The technique can be utilized to characterize scleral globes of normal, high risk and glaucomatous donor tissues in order to provide a better understanding of the biomechanical environment that surrounds the ONH.

## Ethics Statement

Our study was reviewed by University of Pittsburgh Institutional Review Board. It was determined to be exempt from ethics approval and includes no involvement of human subjects according to the federal regulations [§45 CFR 46.102(f)].

## Author Contributions

JV and AA conceived the idea. JV guided AA and HK on the design of the method. HK performed and designed the presented work. AA performed and designed the initial work and writing. RB performed DOE study for material parameters and additional computational work. ET performed S-DIC experiment. KF performed thickness measurement experiment. MD contributed to the final version of the manuscript.

### Conflict of Interest Statement

The authors declare that the research was conducted in the absence of any commercial or financial relationships that could be construed as a potential conflict of interest.
